# Preoperative psychological factors predict talonavicular fusion outcomes in Muller-Weiss disease

**DOI:** 10.3389/fpsyt.2025.1571794

**Published:** 2025-09-30

**Authors:** Xiaocong Liu, Yalei Pan, Shihang Cao, Jun Lu, Junkui Xu

**Affiliations:** ^1^ Honghui Hospital, Xi'an Jiaotong University, Xi'an, Shaanxi, China; ^2^ Co-construction Collaborative Innovation Center for Chinese Medicine Resources Industrialization by Shaanxi & Education Ministry State, Shaanxi University of Chinese Medicine, Xianyang, China

**Keywords:** Muller-Weiss disease, talonavicular joint fusion, mental state, surgical outcomes, relationship

## Abstract

**Objective:**

The aim of this study was to investigate the association between preoperative anxiety and depression and surgical outcome in patients with Müller-Weiss disease (MWD).

**Methods:**

This retrospective study included 41 patients diagnosed with MWD who underwent talonavicular joint fusion at the Foot and Ankle Surgery Department of Xi'an Honghui Hospital between April 2014 and September 2024. Patients were stratified into two groups based on preoperative anxiety and depression symptoms: Group A (symptomatic) and Group B (asymptomatic). Preoperative and final follow-up assessments included the Hospital Anxiety and Depression Scale (HADS), Visual Analogue Scale (VAS) for pain (0–100 mm), and American Orthopedic Foot and Ankle Society (AOFAS) score.

**Results:**

Among 41 patients with complete follow-up, 20 (49%) exhibited preoperative anxiety and depression symptoms. Both groups demonstrated significant improvement in clinical outcomes following talonavicular joint fusion; however, Group A showed inferior overall outcomes compared to Group B. There is a significant negative correlation between AOFAS score improvement and preoperative anxiety levels (r = -0.62, *P* < 0.05).

**Conclusion:**

In patients undergoing talonavicular fusion surgery the presence of high levels of depression and anxiety is associated with inferior post-operative outcomes; moreover the level of anxiety correlates with post-operative improvement in function, emphasizing the importance of addressing psychological factors in treatment planning.

## 1 Introduction

Muller-Weiss disease (MWD) is characterized by chronic dorsal foot pain, compression fracture of the navicular bone, transverse talar protrusion, and morphological changes in the hindfoot, often presenting idiopathically (1). Disease progression leads to foot deformity and debilitating pain (2). The pathogenesis of MWD remains unclear. Navicular osteonecrosis is classified into stages I-V based on the severity of Meary's horn and foot deformity (3). Surgical intervention is typically indicated for patients with stage III disease or above. However, given the absence of a definitive gold standard for MWD treatment, surgery may be necessary for patients who fail to achieve significant improvement after six months of conservative management (4).

Talonavicular joint fusion represents the primary surgical treatment (5). Reade et al. demonstrated that talonavicular joint fusion effectively restores medial column length, increases medial longitudinal arch height, corrects flatfoot deformity, and maintains midfoot and hindfoot stability (6). This procedure also significantly reduces complications such as nonunion and peripheral joint injury (7). Surgical outcomes are influenced by various factors, including comorbidities such as diabetes (8, 9), hypertension (10), and coronary heart disease (11). Additionally, patients' psychological state, including anxiety and depression, has been associated with surgical outcomes (12).

Severe navicular osteonecrosis results in foot deformities, debilitating pain, and functional impairment, significantly affecting daily activities including work, education, and social participation. This can diminish patients' quality of life and increase the likelihood of anxiety and depression, potentially hindering recovery following talonavicular joint fusion. Dydyk et al. found that patients with persistent chronic pain experience elevated levels of anxiety and depression, which negatively impact quality of life and may increase the risk of self-harm (13). Weinerman et al. demonstrated that anxiety and depression are predictive factors for poor surgical outcomes in orthopedic trauma patients (14), with negative psychological states affecting surgical prognosis (15–17). Nathaniel P.M et al. have demonstrated that mental health disorders adversely affect subjective outcomes and complications following total ankle arthroplasty (18). Shihang Cao et al. found that abnormal mental and psychological states such as anxiety and depression before surgery would reduce the surgical prognosis of patients with Hepple V osteochondral lesions of the talus (19).

Multiple studies have established that poor mental health adversely impacts functional outcomes following joint replacement (20–22). Therefore, appropriate psychological interventions may enhance surgical outcomes and improve overall quality of life.

Although surgical treatment for MWD is well-established clinically, the association between preoperative mental state and surgical outcomes remains unclear. This study aims to investigate the association between preoperative anxiety and depression and postoperative pain, functional recovery, and overall surgical outcomes in MWD patients through standardized questionnaire-based assessment. These findings will help elucidate the relationship between preoperative mental state and treatment outcomes, ultimately informing strategies to optimize patient selection and improve surgical efficacy.

## 2 Research methods

This retrospective study received institutional review board approval (approval number 202410012). Data were collected from MWD patients who underwent talonavicular joint fusion at the Foot and Ankle Surgery Department of Xi'an Honghui Hospital between April 2014 and September 2024. All patients provided informed consent.

Inclusion criteria: (1) MWD was diagnosed based on clinical symptoms (pain and tenderness over the navicular and naviculocuneiform joints, with potential medial longitudinal arch collapse in some patients) and weight-bearing anteroposterior and lateral radiographs of the foot; (2) failed conservative treatment for six months with persistent pain; (3) Maceira stage III disease or higher with closed epiphyseal plates; (4) talonavicular joint fusion as the surgical procedure; (5) minimum follow-up of 12 months and the follow-up period was from the surgery to the clinical assessment at the last follow-up. Exclusion criteria: (1) incomplete clinical data; (2) concomitant foot trauma; (3) concurrent foot pathology, including subtalar arthritis, bone tuberculosis, rheumatoid arthritis, or bone tumors; (4) Patients with a history of psychiatric disorders (including bipolar disorder, schizophrenia, depression, anxiety disorders, and other major psychiatric conditions). In this study, patients were contacted via telephone or the WeChat messaging platform.

All patients were treated by the same foot and ankle surgeon using standardized anesthesia and surgical techniques. Eligible patients were identified from medical records and subsequently contacted by telephone to invite participation in the study. Upon consent, baseline demographic and surgical data were extracted from medical records, while current functional outcomes, pain levels, and psychological status were assessed through standardized questionnaires administered during the follow-up contact.

Patient contact information was retrieved from the medical record system, and eligible patients were contacted and invited to participate. Demographic data were collected from enrolled patients, who completed questionnaires assessing subjective symptoms and functional status. The follow-up period was from the surgery to the clinical assessment at the last follow-up.

The Hospital Anxiety and Depression Scale (HADS) assessed anxiety and depression levels at baseline and final follow-up. The HADS comprises two subscales: HADS-A (anxiety) and HADS-D (depression), each containing seven items scored on a four-point scale (0-3). Scores of eight or higher indicate clinically significant anxiety or depression (23, 24). Pain intensity was evaluated using the Visual Analogue Scale (VAS) at baseline and final follow-up. The VAS consists of a 10-cm horizontal line anchored by "no pain" (0 mm) and "worst possible pain" (100 mm), with patients marking their perceived pain level (25). Functional status was assessed using the American Orthopedic Foot & Ankle Society (AOFAS) Midfoot Score at baseline and final follow-up. The AOFAS scoring system quantifies foot health status and monitors treatment progress. Trained evaluators ensured accurate scoring. All questionnaire responses and clinical data were entered into a standardized electronic database and anonymized to ensure accuracy and patient confidentiality.

Patients were stratified into two groups based on preoperative HADS scores: Group A (anxiety or depression symptoms present, score ≥8 on either subscale) and Group B (no anxiety or depression symptoms, score <8 on both subscales).

## 3 Statistical methods

Statistical analysis was conducted using SPSS 27.0 software. The Shapiro-Wilk test was performed to assess normality for all continuous variables. Normally distributed data were presented as mean ± standard deviation (SD), with between-group comparisons performed using independent samples t-tests. Effect sizes were reported as Cohen's d, along with 95% confidence intervals and mean differences. Within-group comparisons utilized paired samples t-tests. Non-normally distributed data were presented as median (first quartile, third quartile) [M (Q1, Q3)]. Between-group comparisons for non-parametric data employed the Mann-Whitney U test, with effect sizes reported as rank-biserial correlation coefficients (r), median differences, and 95% confidence intervals. Categorical variables were described as frequencies and percentages (%), with group comparisons performed using χ² tests.

For patients with anxiety and depression, independent samples t-tests were used to compare psychological status between genders. Pearson correlation analysis was performed to assess relationships between age, postoperative functional improvement, and anxiety/depression scores. All statistical tests were two-tailed with significance set at *P* < 0.05. Data visualizations were created using GraphPad Prism version 10.0.

## 4 Surgical methods

A medial anterior foot incision was made, and the talonavicular joint was exposed layer by layer. The necrotic bone and surrounding osteophytes were removed, and the joint was reduced to the appropriate position. Cancellous bone graft from the tibia/ilium was used, and the talonavicular joint was fixed with hollow nails/micro-plates. Stability of the fusion was confirmed by movement on the platform. C-arm fluoroscopy confirmed proper alignment of the talonavicular joint and internal fixation. The incision was closed layer by layer after irrigation.

A medial longitudinal incision was made over the talonavicular joint, which was exposed through sequential tissue dissection. Necrotic navicular bone and periarticular osteophytes were debrided, and the joint surfaces were prepared for fusion. Anatomical reduction of the talonavicular joint was achieved and maintained with autologous cancellous bone graft harvested from the tibia or ilium. Joint fusion was secured using either cannulated screws or low-profile plates. Construct stability was assessed through manual stress testing. Intraoperative fluoroscopy confirmed appropriate joint alignment and satisfactory hardware positioning. Following copious irrigation, the surgical site was closed in anatomical layers.

## 5 Postoperative rehabilitation protocol

Following surgery, we elevated the affected limb and implemented symptomatic management including anti-inflammatory measures and analgesic therapy. Prophylactic antibiotic therapy was administered for 72 hours postoperatively to prevent surgical site infection, with dressing changes performed every 48 hours. Suture removal occurred on postoperative day 14. Between 6 and 8 weeks postoperatively, patients initiated active and passive range-of-motion exercises for the toes and plantar joints under non-weight-bearing conditions. Partial weight-bearing ambulation commenced at 8–12 weeks postoperatively. Radiographic evaluation was performed at 6, 8, and 12 weeks postoperatively in the outpatient setting. Full weight-bearing ambulation was permitted only after radiographic and clinical evidence confirmed adequate bone healing.

## 6 Imaging data

**Figure 1:**
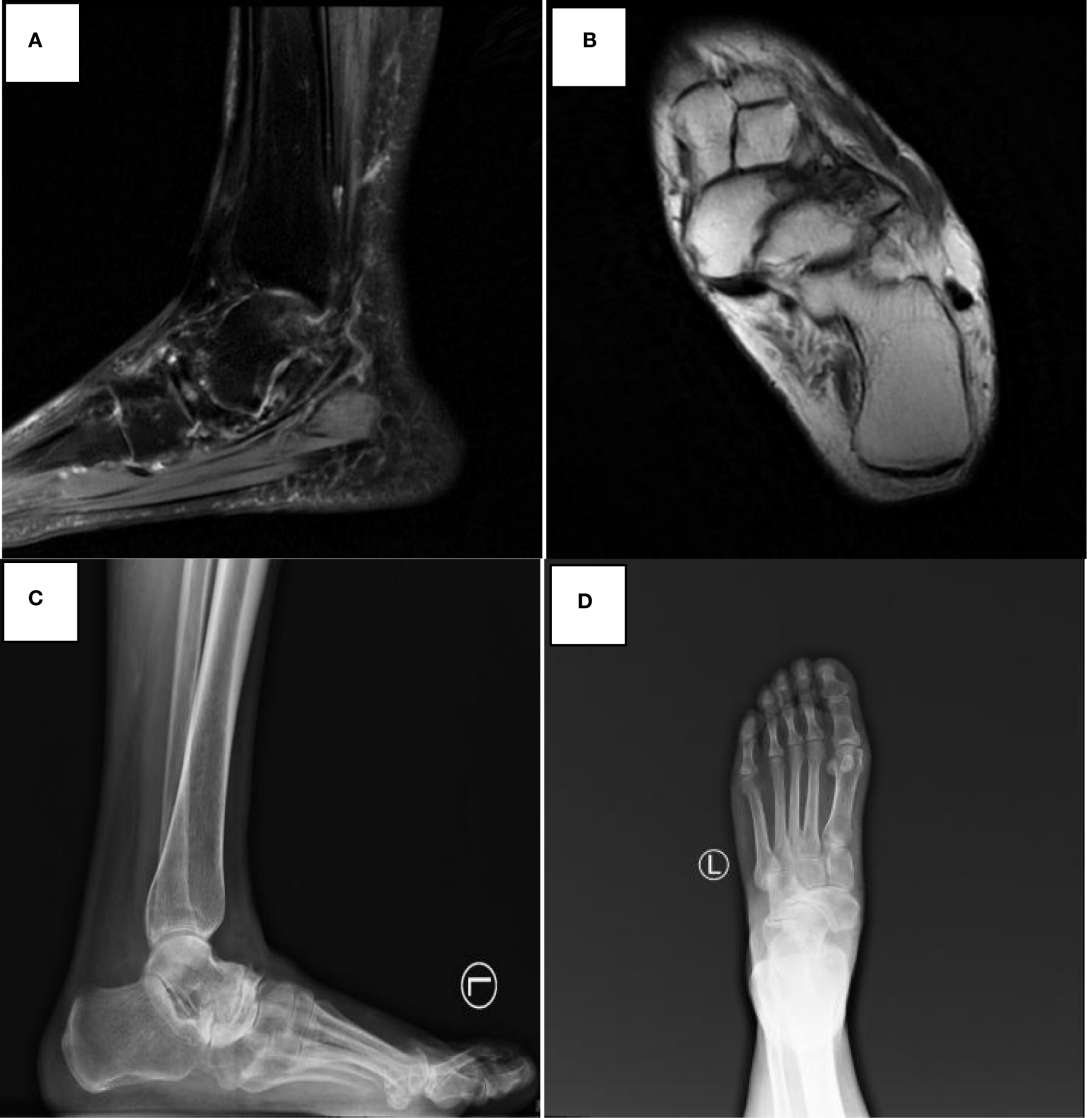
Preoperative imaging of navicular avascular necrosis in Muller-Weiss disease. **(A, B)** Preoperative magnetic resonance imaging demonstrates lateral compression and collapse of the navicular bone with avascular necrosis, subchondral cystic changes, and bone marrow edema, confirming Muller-Weiss disease. **(C, D)** Preoperative weight-bearing radiographs showing characteristic navicular deformity and talonavicular joint incongruity.

**Figure 2:**
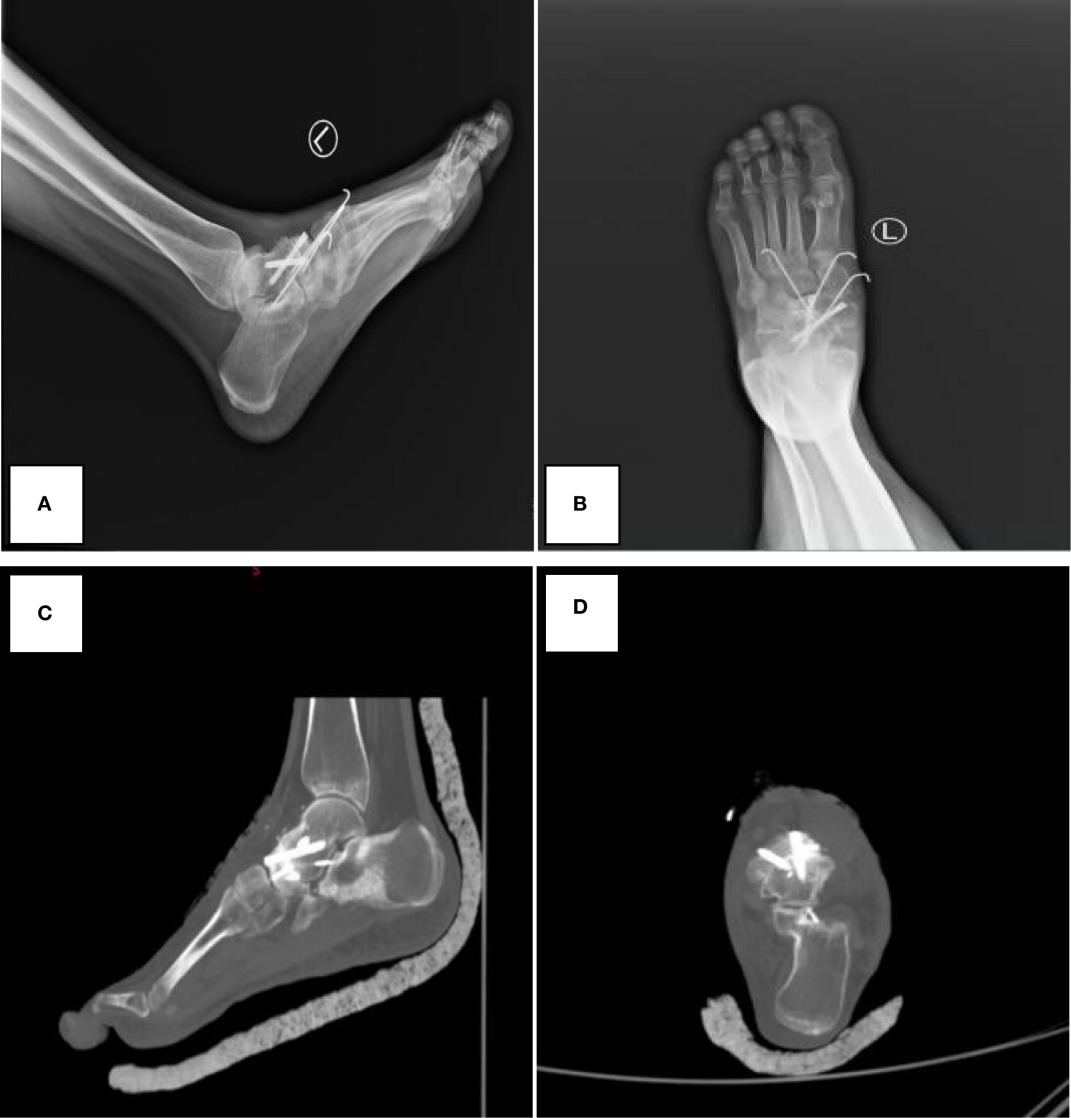
Postoperative imaging demonstrating successful talonavicular joint fusion with hardware *in situ*. **(A, B)** Postoperative X-ray images. **(C, D)** Postoperative CT images. The images demonstrate good alignment of the talonavicular joint and internal fixation.

The imaging data are shown in **Figures 1** and **2**.

## 7 Results

### 7.1 Comparison of baseline data between groups

Of 56 enrolled patients, 41 completed full follow-up and were included in the final analysis. The cohort comprised seven males and 34 females with a median age of 51.00 (40.00, 59.00) years and mean follow-up of 51.83 ± 33.47 months.

Twenty patients demonstrated preoperative anxiety or depression (4 males, 16 females) with mean follow-up of 57.25 ± 32.30 months. Twenty-one patients without preoperative anxiety or depression (3 males, 18 females) had mean follow-up of 46.67 ± 34.53 months.

**Figure 3:**
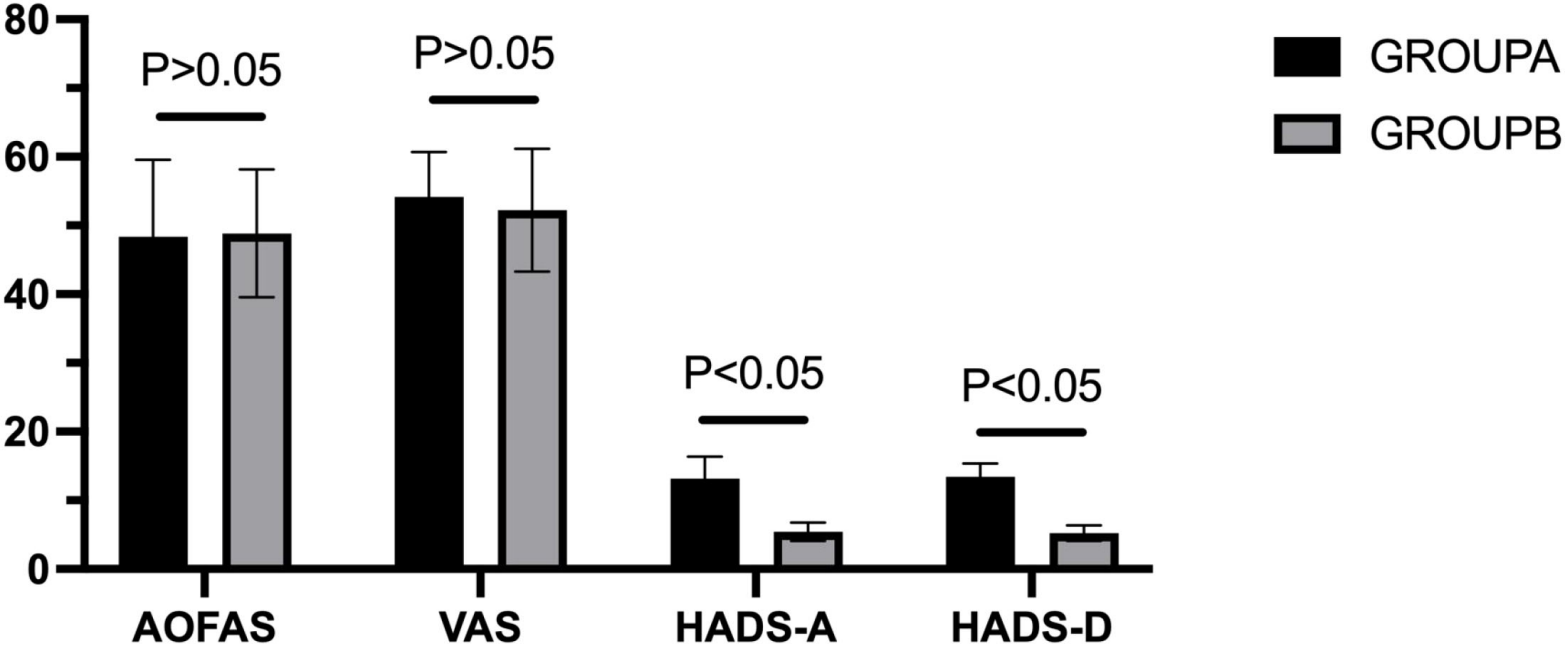
Comparison of preoperative evaluation indicators between groups.

No significant between-group differences were observed for demographic variables, follow-up duration, or preoperative AOFAS and VAS scores (*P* > 0.05). As expected, preoperative HADS-A and HADS-D scores differed significantly between groups (*P* < 0.01). Detailed baseline characteristics are presented in **Table 1**, with preoperative score comparisons shown in **Figure 3**.

**Table 1:** Baseline characteristics of patients (Mean ± SD/Median [Q1, Q3]).

Variables	Group A(n=20)		Group B (n=21)		*t/Z/*χ²	*P*	Group difference (95% CI)	Effect size (95% CI)
	Male	Female	Male	Female				
Sex	4(20.00)	16(80.00)	3(14.28)	18(85.71)	0.70	0.47	–	–
Age	48.00(25.00,55.50)		54.00(43.50,62.50)		-1.71	0.09	Med diff:-7.50(-17.00,1.00)	r:-0.37(-0.66,-0.02)
Follow-uptime	57.25 ± 32.30		46.67 ± 34.53		1.01	0.32	MD:10.58(-10.56,31.73)	d:0.32(-0.30,0.93)
AOFAS	48.35 ± 11.20		48.86 ± 9.29		-0.16	0.88	MD:-0.51(-6.99,5.98)	d:-0.05(-0.66,0.56)
VAS	54.15 ± 6.57		52.24 ± 8.93		0.78	0.44	MD:1.91(-3.06,6.88)	d:0.24(-0.37,0.86)
HADS-A	14.00(10.25,16.00)		6.00(4.00,7.00)		-5.51	0.00	Med diff: 8.00(6.00,10.00)	r:-0.86(-0.93,-0.73)
HADS-D	14.00(12.00,15.00)		5.00(4.00,6.00)		-5.52	0.00	Med diff: 8.00(7.00,9.00)	r:-0.86(-0.94,-0.75)

MD, Mean Difference; Med diff, Median difference (Hodges-Lehmann estimator).

d, Cohen's d; r, Rank-biserial correlation.

### 7.2 Pre- and postoperative outcome measures

Following talonavicular joint fusion, both groups demonstrated significant improvements in all outcome measures: AOFAS scores, VAS pain ratings, and HADS-A and HADS-D scores (*P* < 0.01). Detailed results are presented in **Table 2**.

**Table 2:** Comparison of preoperative and final follow-up scores between groups (Mean ± SD).

Variables		Pairing		*t*	*P*	Group difference (95% CI)	Effect size (95% CI)
		Before operation	Last follow-up				
Group A(n=20)	AOFAS VAS HADS-A HADS-D	48.35 ± 11.20	70.50 ± 8.28	-8.70	0.00	MD:-22.15(-27.51,-16.79)	d:11.45(-2.68,-1.17)
		54.15 ± 6.57	18.15 ± 5.75	15.99	0.00	MD:36.00(31.29,40.71)	d:3.58(2.36,4.78)
		14.00(10.25,16.00)	6.00(6.00,7.00)	-3.93	0.00	Meddiff:-6.50(-8.00,-5.00)	r:-0.67(-0.81, -0.49)
		14.00(12.00,15.00)	6.25 ± 1.52	-3.94	0.00	Meddiff:-7.50(-8.50,-6.00)	r:-0.67(-0.81, -0.50)
Group B(n=21)	AOFAS VAS HADS-A HADS-D	48.86 ± 9.29	86.24 ± 6.11	-16.08	0.00	MD:-37.38(-42.23,-32.53)	d:10.65(-4.66,-2.34)
		52.24 ± 8.93	4.67 ± 2.03	24.66	0.00	MD:47.57(43.55,51.60)	d:8.84(3.67,7.08)
		6.00(4.00,7.00)	1.00(0.50,2.00)	-3.97	0.00	Meddiff:-4.00(-5.00,-3.50)	r:-0.67(-0.81, -0.50)
		5.00(4.00,6.00)	2.00(1.00,3.00)	-4.07	0.00	Meddiff:-3.00(-4.00,-3.00)	r:-0.68(-0.82, -0.51)

MD, Mean Difference; Med diff, Median difference (Hodges-Lehmann estimator).

d, Cohen's d; r, Rank-biserial correlation.

### 7.3 Between-group postoperative outcomes

**Figure 4:**
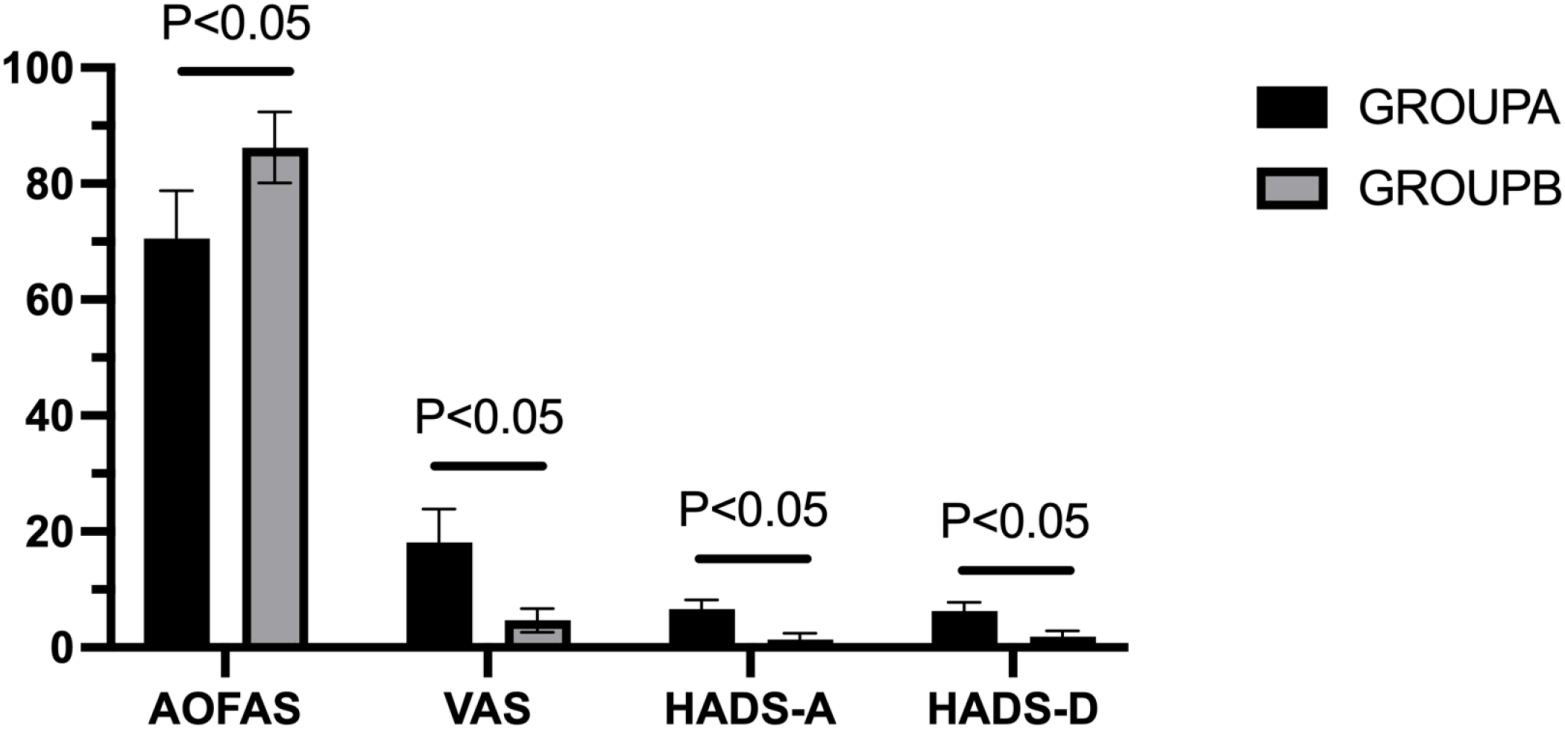
Comparison of postoperative evaluation indicators between groups.

Significant between-group differences were observed in all postoperative outcome measures (*P* < 0.01). Patients without preoperative anxiety or depression (Group B) achieved superior functional and psychological outcomes compared to those with preoperative anxiety or depression (Group A), indicating that baseline psychological status significantly influences postoperative recovery (**Figure 4**). Detailed postoperative comparisons are presented in **Table 3**.

**Table 3:** Comparison of postoperative evaluation indicators between groups (Mean ± SD).

Variables	AOFAS	VAS	HADS-A	HADS-D
Group A(n=20)	70.50 ± 8.28	18.15 ± 5.75	6.00(6.00,7.00)	6.25 ± 1.52
Group B(n=21)	86.24 ± 6.11	4.67 ± 2.03	1.00(0.50,2.00)	2.00(1.00,3.00)
*t*	-6.95	9.91	-5.53	-5.45
*P*	0.00	0.00	0.00	0.00
Group Difference (95% CI)	MD:-15.74(-20.32,-11.16)	MD:13.48(10.67,16.30)	Meddiff:5.00(4.00,6.00)	Meddiff:4.00(4.00,5.00)
Effect Size (95% CI)	d:-2.17(-2.94,-1.38)	d:4.27(2.22,4.08)	r: -0.86 (-0.93, -0.75)	r: -0.85 (-0.92, -0.73)

MD, Mean Difference; Med diff, Median difference (Hodges-Lehmann estimator).

d, Cohen's d; r, Rank-biserial correlation.

### 7.4 Comparison of pre- and postoperative changes in AOFAS, HADS-A, HADS-D, and VAS scores between groups

Statistical analysis demonstrated a statistically significant difference in AOFAS score changes between the two groups (*P* < 0.01), whereas no significant difference was observed in VAS score changes (*P* = 0.96). The differences between preoperative and final follow-up scores are presented in **Table 4**.

**Table 4:** Comparison of changes in evaluation indicators between groups before and after surgery (Mean ± SD).

Variables	AOFAS changes	VAS changes	HADS-A changes	HADS-D changes
Group A(n=20)	22.15 ± 11.45	36.00 ± 10.07	6.50 ± 3.35	7.15 ± 2.20
Group B(n=21)	37.38 ± 10.65	47.57 ± 8.84	3.86 ± 1.20	3.00(3.00,4.00)
*t/Z*	-4.29	-3.92	-4.12	-4.63
*P*	0.00	0.00	0.00	0.00
Group Difference (95% CI)	MD:-14.56(-21.43,-7.69)	MD:-11.57(-17.55,-5.59)	MD:3.00(2.00,5.00)	Meddiff:4.00(3.00,5.00)
Effect Size (95% CI)	d:-1.34(-2.01,-0.65)	d:-1.22(-1.89,-0.55)	d:1.71(1.14,2.28)	r:-0.72 (-0.83, -0.58)

MD, Mean Difference; Med diff, Median difference (Hodges-Lehmann estimator).

d, Cohen's d; r, Rank-biserial correlation.

### 7.5 Correlation analysis between psychological status and clinical variables in group A

**Figure 5:**
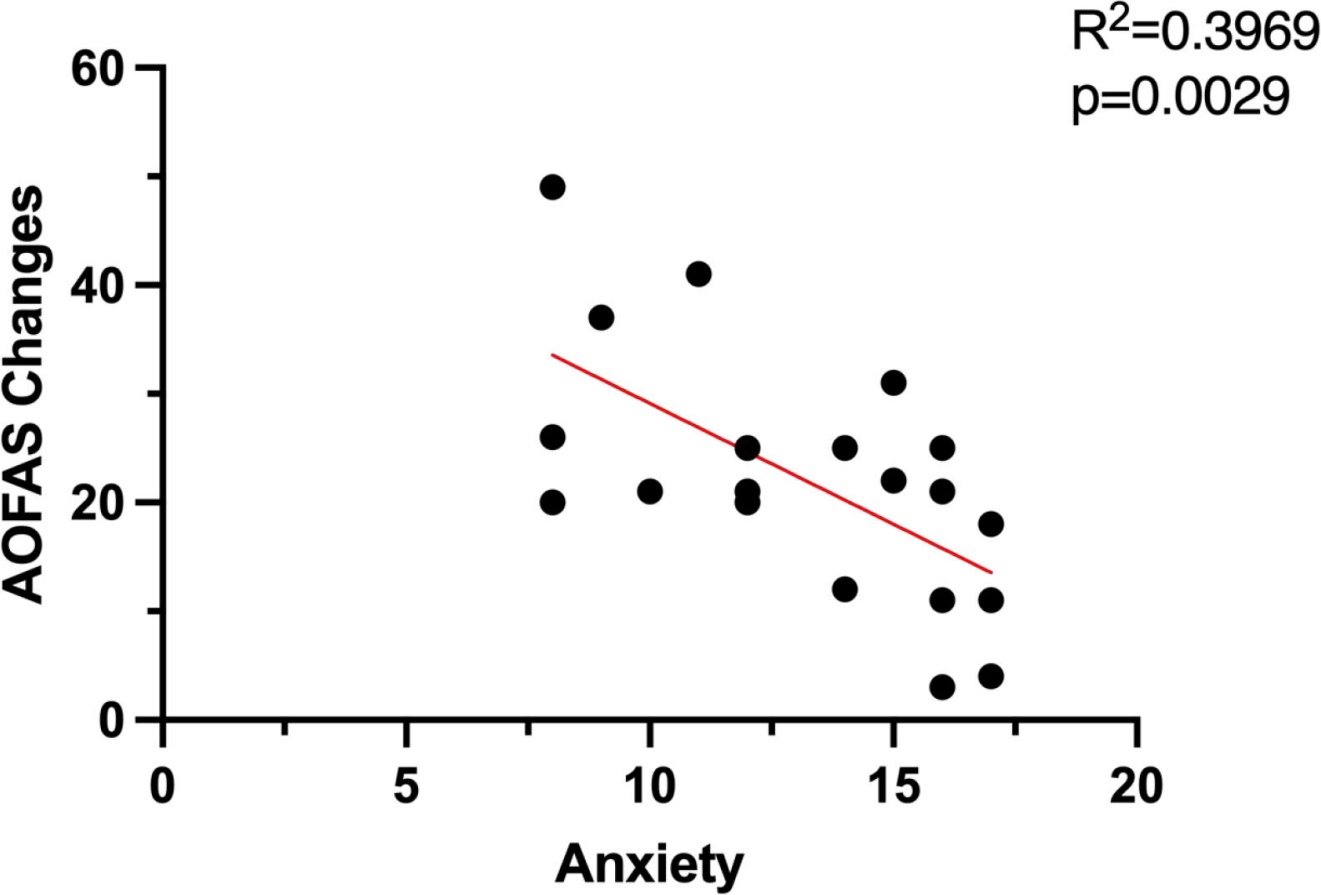
Correlation between postoperative improvement in AOFAS score and preoperative anxiety in group A.

In Group A, no significant correlations were identified between age, gender, and preoperative anxiety or depression scores. However, a significant negative correlation was observed between AOFAS score improvement and preoperative anxiety levels (r = -0.62, *P* < 0.05), as shown in **Figure 5**. Preoperative depression levels demonstrated no significant correlation with functional outcomes in this analysis (r = 0.12, *P* > 0.05). Comprehensive results are presented in **Tables 5** and **6**.

**Table 5:** Correlation between age, gender, and preoperative anxiety and depression in group A.

Variables		Anxiety	Depression
Age	*r* P	0.48	-0.24
		0.05	0.32
Sex	*r* P	0.15	-0.28
		0.52	0.24

**Table 6:** Correlation between postoperative improvement in AOFAS score and preoperative anxiety and depression in group A.

Variables		Anxiety	Depression
AOFAS difference	*r* P	-0.62	0.12
		0.00	0.62
VAS difference	*r* P	0.05	-0.01
		0.83	0.97

## 8 Discussion

Midfoot deformity with pain, also known as Brailsford disease (MWD), predominantly affects middle-aged women (40–60 years) and has a relatively low incidence (26). Cotchett et al. reported that negative emotions frequently accompany foot and ankle pain (27), adversely impacting surgical outcomes (28). According to Sharma et al., referral of patients with high-risk psychosocial factors could be essential to improve postoperative outcomes and satisfaction (29).

This study demonstrated significant postoperative improvements in functional activity, pain levels, and psychological status in both groups following talonavicular (TN) fusion. Hu et al. reported increased AOFAS scores from 43.5 ± 12.4 to 83.8 ± 9.1 and decreased VAS scores from 7.3 ± 1.5 to 1.0 ± 0.4 after TN fusion (30). Similarly, Liang Lu et al. found that in patients with MWD, AOFAS scores improved from a preoperative value of 43.4 ± 16.1 to 85.3 ± 6.2, further confirming the effectiveness of this surgical approach in enhancing foot and ankle function in MWD patients (31). The conclusions in this study are all greater than the minimal clinically important difference (MCID) in the relevant research literature, further confirming the effectiveness of this surgical method (32, 33). Our analysis of psychological status revealed significant improvements in postoperative HADS-A and HADS-D scores, with no adverse events such as wound infection or nonunion observed during follow-up. These findings suggest that TN fusion is an effective treatment modality for MWD and all subjects experience an improvement that is clinically and statistically significant, as seen by other authors.

Postoperative VAS and AOFAS scores differed significantly between the two groups. Preoperative anxiety or depression was associated with suboptimal pain management and functional recovery. This finding aligns with previous research indicating that poor preoperative psychological status is associated with diminished functional recovery and increased postoperative pain (34). Agarwalla et al. (35) and Lai (36) similarly reported that depression and poor mental health negatively affected outcomes after biceps tendon fixation and bunion surgery, respectively. Therefore, it appears that preoperative psychological status could influence postoperative outcomes.

The mean improvement in AOFAS scores was 22.15 ± 11.45 and the mean reduction in VAS scores was 36.00 ± 10.07 in Group A. These improvements were significantly smaller than those observed in Group B (AOFAS: 37.38 ± 10.65; VAS: 47.57 ± 8.84) (*P* < 0.05). Baseline demographic and clinical data (gender, age, preoperative AOFAS and VAS scores) showed no significant differences between groups. However, significant differences emerged in postoperative AOFAS and VAS scores despite both groups receiving identical surgical interventions. Although postoperative pain is common, patients with preoperative anxiety or depression experienced inferior functional outcomes and elevated pain levels compared to those without these conditions (37). This phenomenon may be attributed to reduced participation in postoperative rehabilitation programs. While preoperative AOFAS and VAS scores did not differ significantly between groups, Group A exhibited significantly higher postoperative VAS scores and lower AOFAS scores with greater variability than Group B (*P* < 0.05). Both groups demonstrated significant improvements in postoperative HADS-A and HADS-D scores. However, given that Group B had lower baseline anxiety and depression scores, their improvement was less pronounced than Group A. These findings suggest that preoperative psychological status significantly impacts postoperative pain management and functional recovery, as reflected in AOFAS and VAS scores.

In Group A patients, no significant correlations were identified between age or gender and levels of anxiety or depression. This finding may be attributed to the limited sample size and unequal gender distribution in this study. Among Group A patients, a significant negative correlation was observed between postoperative functional improvement and preoperative anxiety levels, whereas no significant correlation was found with preoperative depression. We hypothesize that elevated preoperative anxiety levels may impair patients' ability to cooperate fully with medical personnel before surgery, thereby compromising their confidence in the surgical team. Previous studies have demonstrated that patients with high preoperative anxiety often experience poor sleep quality, and the severity of sleep disorders is significantly negatively correlated with postoperative physical function (38). Patients with compromised sleep quality are at increased risk for fractures, falls, and other adverse events (39). Excessive activity of the affected limb may precipitate secondary injuries, thereby compromising surgical outcomes. Relevant studies have found that patients who experience anxiety before surgery often have higher levels of stress hormones such as cortisol, epinephrine, and norepinephrine, which in turn affects the surgical prognosis of the patients (40). Conversely, no significant correlation was observed between preoperative depression and postoperative prognosis. This may be because preoperative depression primarily intensifies patients' negative emotions without directly affecting physical activity levels. This study highlights the importance of assessing anxiety in patients undergoing surgery for MWD and the HADS questionnaire can be a useful tool for this purpose. This would allow for the implementation of strategies aimed at reducing anxiety levels, such as deep breathing (41), muscle relaxation (42), music therapy (43, 44), and guided imagery (45). What's more, it's possible to reduce the level of anxiety and depression of patients by proper nursing measures just like what Emily L. Zale et al. have reported (46). We also recommend that particular attention should be given to evaluating the psychological status of MWD patients, especially anxiety levels, to develop individualized treatment plans that enhance the clinical effectiveness of surgical intervention.

This study has several limitations: (1) a relatively small sample size; (2) subjective assessment methodologies for foot function and pain evaluation; (3) the potential influence of confounding variables such as occupation and living conditions on patient outcomes; (4) the impact of socioeconomic factors on foot and ankle functional recovery. Furthermore, the retrospective study design limits the ability to establish definitive causal relationships between variables; (5)variability in the timing of follow-up assessments may have introduced measurement bias. Future investigations should incorporate larger sample sizes to minimize the influence of confounding factors and, when feasible, conduct prospective studies to validate these findings.

## 9 Conclusion

In patients undergoing talonavicular fusion surgery the presence of high levels of depression and anxiety is associated with inferior post-operative outcomes; moreover the level of anxiety correlates with post-operative improvement in function. The findings of this study suggest that clinical management of similar patients should incorporate appropriate psychological interventions to mitigate preoperative anxiety and psychological distress. This comprehensive approach may optimize surgical outcomes, facilitate functional recovery, and ultimately improve patients' overall quality of life.

## Data Availability

The raw data supporting the conclusions of this article will be made available by the authors, without undue reservation.
